# Discourse-based psychological intervention alleviates perioperative anxiety in patients with adolescent idiopathic scoliosis in China: a retrospective propensity score matching analysis

**DOI:** 10.1186/s12891-023-06438-2

**Published:** 2023-05-26

**Authors:** Luosha Bi, Chengjun Pan, Jiaxing Li, Jiahui Zhou, Xiangyu Wang, Shiqi Cao

**Affiliations:** 1 Department of College English Teaching and Research, Capital Normal University, Beijing, People’s Republic of China; 2 Medical Unit, Unit 61016 of the People’s Liberation Army, Beijing, People’s Republic of China; 3 Department of English Teaching, Beijing No.50 Middle School, Beijing, People’s Republic of China; 4 College of Teacher Education, Capital Normal University, Beijing, People’s Republic of China; 5 Department of Pain Medicine, the First Medical Center, Chinese PLA General Hospital, Fuxing Rd. 28, Haidian District, Beijing, People’s Republic of China; 6 Department of Orthopaedics, the Fourth Medical Center, Chinese PLA General Hospital, Beijing, People’s Republic of China; 7 Orthopaedics of TCM Senior Department, the Sixth Medical Center, Chinese PLA General Hospital, Beijing, People’s Republic of China

**Keywords:** Scoliosis, Anxiety, Pain, Personal satisfaction, Adolescent psychology, Retrospective study

## Abstract

**Purpose:**

To evaluate the effectiveness of a discourse-based psychological intervention on perioperative anxiety, pain and life satisfaction of patients with AIS.

**Methods:**

Between April 2018 and February 2021, 116 consecutive patients with AIS undergoing corrective surgery were enrolled in this study, including 51 with personalized psychological intervention (intervention group, IG) and 65 without (control group, CG). After propensity score matching (PSM), patient characteristics, perioperative scores of anxiety and life satisfaction, measured by values of Generalized Anxiety Disorder 7-item Scale (GAD-7) and Life Satisfaction Index Z scale (LSIZ), were recorded. Mixed linear models were used to estimate the influence of intervention group and time of measurement, as well as their interactions, in anxiety and life satisfaction. Data on post-surgical pain in both groups was also collected and analyzed.

**Results:**

After PSM, a total of 90 patients (IG, n = 45; CG, n = 45) were enrolled in this study, and the 2 groups were comparable in patients’ demographic and baseline characteristics. There were no pre-intervention between-group differences in the degree of anxiety (IG: 3.98 ± 3.27 vs. CG: 3.93 ± 3.20, p = .948, Cohen’s d = 0.015), and life satisfaction (IG: 6.56 ± 1.70 vs. CG: 6.67 ± 2.09, p = .783, Cohen’s d = -0.058). After surgery, participants in both IG and CG showed improved the levels of anxiety (GAD-7: IG 2.18 ± 1.21; CG 2.87 ± 2.00) and life satisfaction (LSIZ: IG 9.84 ± 2.09; CG 9.02 ± 2.15). A stratified analysis of patients with generalized anxiety disorder showed improved anxiety (GAD-7: IG 3.50 ± 1.22 vs. CG 6.80 ± 2.05, p = .017, Cohen’s d = -1.956) and lower pain level (VAS: IG 4.50 ± 1.76 vs. CG 7.00 ± 1.00, p = .017, Cohen’s d = -1.747) in the IG than the CG after surgery.

**Conclusions:**

Discourse-based psychological intervention before surgery can improve perioperative anxiety and life satisfaction, and postoperative painful condition, especially for patients with high-leveled pre-surgical anxiety.

## Background

Adolescent idiopathic scoliosis (AIS) is a three-dimensional deformity of the spine, which occurs in adolescents. The deformation, with the major Cobb ≥ 10°, is usually associated with the asymmetry of torso, shoulders and waist, combined with the rib hump [1, 2]. For those with a Cobb ≥ 40°, surgical intervention is usually recommended, as the deformity would impair patients’ quality of life [3]. According to previous studies, over 50% of AIS patients were recognized to experience surgery-related anxiety or significant psychological stresses before corrective operation [4]. As preoperative anxiety was proved to have close association with many postoperative issues, such as pains, sleeping disturbances, eating disorders, separation anxiety, it was strongly recommended to effectively improve preoperative negative emotions of adolescents [5, 6].

To date, many strategies have been proposed on the alleviation of surgery-related anxiety, such as knowledge level improvement [7, 8], participation in decision-making [9], comprehension in informed consent for surgical procedures [10]. However, they were normally designed for adults, and many related knowledge and instructions were beyond the scope of adolescents’ cognition and comprehension [6]. There were several interventions designed specifically for adolescents, such as parental presence and music therapy, but they were either ineffective in high-level stress situations or highly hospital-policy depended [11, 12].

In recent years, a more effective way was highlighted, for healthcare providers, to alleviate adolescent patients’ anxiety, that is, to communicate with adolescents so as to increase their understanding of adolescents’ psychological processes and improve the way how adolescents perceive the disease and surgery [13]. Since the 1990s, there has been evidence that discourse-based psychological intervention was beneficial to understanding and treating emotional disorders, such as anxiety and depression [14, 15]. To date, psychological intervention via discourse analysis has been applied to the alleviation of psychological distress caused by many chronic diseases, such as chronic obstructive pulmonary disease, diabetes, rheumatoid arthritis, coronary heart disease [16–18] and pediatric disabilities [19]. Besides, it was shown that for adults undergoing surgery under general anesthesia, this psychological intervention was beneficial for postoperative pain and other surgery-related outcomes as well [20].

Although previous researches suggested that discourse-based psychological intervention should be effective in reducing patients’ preoperative anxiety [20], to the best of our knowledge there was no research on the correlation between discourse-based psychological intervention and perioperative anxiety of AIS. The present study was aimed to evaluate the effectiveness of psychological intervention in improving perioperative anxiety, pain and life satisfaction of AIS patients who underwent surgery through discourse analysis.

## Materials and methods

### Participants and study design

A retrospective analysis of prospectively collected data was conducted in the Department of Orthopaedics spine unit in authors’ hospital. A total of 116 consecutive patients (15 males and 101 females) with AIS undergoing corrective surgery between April 2018 and February 2021 were analyzed in this study, including patients who received personalized psychological intervention (intervention group, IG; n = 51), and who didn’t receive personalized psychological intervention (control group, CG; n = 65). Inclusion criteria were: admission to the Orthopaedic ward in authors’ hospital for primary corrective operation, Cobb angle of the major curve ≥ 40°, age between 11 and 18 years at the time of admission, absence of cord, vertebral or conus anomaly, and proficiency in Chinese. Exclusion criteria included: inability to independently complete the procedures involved in the interview and intervention, cognitive or functional restriction, and history of spinal surgeries or other invasive treatments, or severe psychiatric disorders, such as schizophrenia, bipolar affective disorder, schizoaffective disorder, or paranoiac psychosis. Basic demographics (age, gender, family income and parents’ higher educational level), previous surgeries, psychiatric history, clinical assessments were collected from participants’ clinical records. The protocol of this study was approved by the hospital administration and ethics committee.

In order to make this study as close as possible to the randomized clinical trial environment, propensity score matching (PSM) was used to match the 2 groups of patients in the proportion of 1:1, so as to achieve the comparability of potential confounding variables between IG and CG. After PSM, the same sample size in IG (n = 45) and CG (n = 45) was established.

### Procedure

#### Interview

Both groups received routine preoperative patient education, including standardized information on surgical procedures by nurses. Additionally, each patient in the IG was given a qualitative semi-structured one-on-one informal interview lasting 15 min. The interviewer asked several open-ended questions, addressing specific topics on participants’ understanding and experiences of having AIS and how they managed the anxiety caused by AIS and surgery. In the interview, participants were encouraged to actively participate and speculate on the primary cause of anxiety as well as their true feelings and reactions to the anxiety in various situations. In this study, interviews, data collection and interventions were conducted in a quiet office in the hospital by medical staffs who have received professional training on psychology and discourse analysis.

#### Discourse analysis

As a way of analytic process, discourse analysis was frequently used to analyze the form of text (i.e. interview transcripts in this study) and understand the construction of self as well as the interaction of meaning within a particular social context [21]. In discourse analysis, speakers’ language was not taken as neutral transmitter of events or experiences; instead, people tended to present their speech rhetorically and achieve certain social actions, such as argumentation, request, apology, appreciation, or blame. Discourse analysts’ duty was to “identify the social actions being performed and the possible function they serve by examining these consequences” [22].

With permission, all participants’ interviews in this study were audiotaped and transcribed verbatim by medical staffs having access to technical support from the qualified graduate students majoring in linguistics. Transcripts were read and divided into units of meaning at least two times to ensure the accuracy during transcription. After that, the data was coded and abstracted into themes, as shown in Table 1. Based on the transcripts, discourse analysis was applied to analyze the participants’ usage of different expressions and sentence structures. Rhetorical strategies (such as metaphor, contrast and paradox), tones, ellipsis and pauses were also paid attention in the analysis. Experiences of anxious and depressive ideation and behavior, and the related meanings and actions were of particular importance.

**Table 1:** Examples of a unit of meaning, code and theme

No.	Meaning unit	Code	Theme
1.	‘[…] *wo bu zhidao zenme shuo…emm…jiushi…(pause)…wo juede wode yangzi hen nankan…wode houbei bu haokan…wo hen zaiyi tongxuede yanguang…wo bu tai yuanyi gen tongxue wan le…ganjue hen diuren….’* NO. 15 En. I don’t know how to say. I feel like I look ugly because of my back. I care about others’ opinions on my appearance very much. I don’t want to play with my classmates. I feel embarrassed.	I look ugly and feel embarrassed because of the deformity.	Aesthetics anxiety
2.	‘[…] *shouxian hen qingxing de shi wo houbei de wanqubianxing meiyou name mingxian… danshi… dangwo yudong de shihou…emm…youqi jinxing julie yundong de shihou wode houbei hui teng…lingwai youxie dongzuo wo ye zuobudao…bi ru wanyao qu goujiaozhi…wo gandao youxie jusang he jiaolv…*’ No. 42 En. First of all, I feel so lucky that my back deformity is not every evident. What annoys me much is that my back hurts when I do exercise, especially strenuous exercise. What’s more, I am not able to do some sports movements, like bending and touching toes. I feel a bit frustrated and anxious.	My back hurts and I cannot do some sports movements. I feel frustrated and anxious.	Activity participation anxiety

The intervention was conducted based on the discourse analysis before the corrective operation, aiming to provide patients with a positive perspective to the disease and the forthcoming operation. The intervention lasted 30 min and consisted of two phases: (1) in the initial phase the participant was lead empathically to identify their psychological and physical symptoms of anxiety based on the transcript, and then they would receive affirmative response from the consultant on these emotions and experiences; (2) in the second phase participant’s concerns and worries about surgery and AIS were highlighted and received explanation; besides, coping strategies and the concept of the “normal range” in health and beauty were conveyed to participants based on the their positive and negative experiences or feelings, so as to reduce their judgmental attitude [23] and anxiety.

### Data collection

The demographic and clinical data was recorded after patients’ decisions for surgery. Effect sizes of anxiety, life satisfaction, and pain were measured according to Cohen’s d calculated by mean and standard deviation values of Generalized Anxiety Disorder 7-item Scale (GAD-7), Life Satisfaction Index Z scale (LSIZ), and Visual Analogue Scale (VAS), respectively. The baseline data of GAD-7 and LSIZ were measured on the day of admission to the hospital (t0). Individual interviews were conducted at the same day of admission (t0) and interventions took place on the following day of admission. As the primary outcome of this study, levels of anxiety and life satisfaction were collected on 1-day before surgery (t1) and 1-day before discharge (t2), i.e., an average 5.62 days after surgery. Besides, patients’ levels of pain were also measured by VAS at t2.

### Instruments

The GAD-7 is a 7-item self-report measure assessing anxiety with the total scores range between 0 and 21. There are 7 crucial anxiety symptoms during the last 2 weeks concerned in the questionnaire, which can be categorized into 4 groups, i.e., minimal (0–4), mild (5–9), moderate (10–14), and severe (≥ 15). Participants with the scores over 10 are considered to have generalized anxiety disorder [24]. The VAS was originally applied to measure mood disorders in psychology and later has been widely used as a tool for pain intensity measurement since the mid-1960s [25]. A 10-centimeter horizontal or vertical line (0–10: from “no pain at all” to “worst pain ever”) is introduced in this scale to allow the participants to evaluate their pain level. Participants are required to make marks according to their perceptions, and the distance from the left endpoint to the mark is measured in mm. The LSIZ was initially developed for the elderly and later modified for adolescents by rephrasing some of the questions with more appropriate for the youth [26]. The modified scale contains 13 agreement-disagreement questions, with the total score ranging from 0 (the lowest satisfaction) to 13 (the highest satisfaction).

### Statistical methods

All data were analyzed using Statistical Package for the Social Sciences, version 26.0 (SPSS, Chicago, IL). The statistical analysis was conducted by an independent statistician who was not directly involved in the study.

Within each group, propensity scores were computed using multivariable logistic regression models. Covariates that were unbalanced between treatment groups prior to matching were included in propensity scores. These covariates might have an impact on both the likelihood that patients would receive the treatment of interest and the outcome of interest. Multiple patient characteristics were among these variables. A 1:1 nearest-neighbor algorithm was used for matching based on propensity scores incorporating various sets of covariates, either with a caliper width of 0.25 (intervention group) or without one (control group). Based on the distribution of propensity scores that was the most evenly distributed and the best balance of individual covariates between the two groups in analysis, the approach that produced the best-matched cohort was determined.

Quantitative data were reported as mean ± standard deviation, and categorical data as frequency and percentage. Analyses of the primary outcomes were undertaken using mixed linear models, where intervention group, time of measurement, and their interaction were set as fixed effect, while participants as random effect. Variance components were used to control random error, and analyze the associations between interventions and variables. The differences between IG and CG were analyzed with t-test for normally distributed variables or with Wilcoxon rank sum test for variables that were not normally distributed. Chi-square or Fisher’s test were applied for categorical variables. A p values < 0.05 were considered statistically significant.

## Results

### Demographic and clinical characteristics

Between April 2018 and February 2021, a total of 116 consecutive patients (15 males and 101 females) with AIS undergoing corrective surgery were analyzed in this study, including 51 with IG and 65 with CG. After PSM, a total of 90 patients were enrolled in this study, and the details of demographic and clinical characteristics of participants before and after propensity score matching are shown in Table 2. There were no significant differences in characteristics of patients between the 2 groups (p > .05). A total of 12 male and 78 female patients were enrolled in our study (6 males and 39 females for the IG and 6 males and 39 females for the CG). Patients’ average years of age were 15.00 ± 1.65 in the IG, and 14.98 ± 1.67 in the CG. The mean Cobb angle of the major curve was 52.07 ± 8.40° in the IG, and 53.91 ± 8.86° in the CG. No statistically significant differences were tested in sex, age, Cobb angle of the major curve, family income, and parents’ higher education level between the IG and the CG after PSM.

**Table 2:** Comparison of demographic and clinical characteristics of participants before and after propensity score matching in the 2 Groups

Characteristics	Total set			Matched set		
	IG (n = 51)	CG (n = 65)	P	IG (n = 45)	CG (n = 45)	P
**Age (years)**	14.94 ± 1.71	15.14 ± 1.78	0.549	15.00 ± 1.65	14.98 ± 1.67	0.950
**Gender**			0.740			1.000
Female	45	56		39	39	
Male	6	9		6	6	
**Cobb angle of the major curve(°)**	52.33 ± 8.82	54.58 ± 9.46	0.193	52.07 ± 8.40	53.91 ± 8.86	0.314
**Income**			0.611			0.480
< 10,000 CNY/month	36	43		31	34	
≥ 10,000 CNY/month	15	22		14	11	
**Education**			0.125			0.788
Junior high school	10	21		9	8	
Senior high school or Higher	41	44		36	37	

### Clinical outcomes

Clinical outcomes for all patients were evaluated by GAD-7, VAS, and LSIZ, as shown in Table 3. There were no pre-intervention between-group differences in the degree of anxiety (IG: 3.98 ± 3.27 vs. CG: 3.93 ± 3.20, p = .948, Cohen’s d = 0.015), and life satisfaction (IG: 6.56 ± 1.70 vs. CG: 6.67 ± 2.09, p = .783, Cohen’s d = -0.058). After psychological intervention via discourse analysis, an improvement of anxiety (GAD-7: 3.98 ± 3.27 to 3.47 ± 2.58, Cohen’s d = 0.173) and life satisfaction (LSIZ: 6.56 ± 1.70 to 8.27 ± 1.94, Cohen’s d = -0.938) was noted at t1 in the IG, but not in the CG (GAD-7: 3.93 ± 3.20 to 4.04 ± 3.08, Cohen’s d = -0.035 and LSIZ: 6.67 ± 2.09 to 6.62 ± 2.08, Cohen’s d = 0.024, respectively). After surgery at t2, participants in both IG and CG showed improved the levels of anxiety (GAD-7: IG 2.18 ± 1.21; CG 2.87 ± 2.00) and life satisfaction (LSIZ: IG 9.84 ± 2.09; CG 9.02 ± 2.15) comparing to t0 and t1. As is listed in Table 4, for both anxiety and life satisfaction, the differences of both t0 and t1 to t2 were significant (p < .001). However, when we focused on the differences caused by the group difference of IG to CG, the group difference couldn’t affect the degree of anxiety and life satisfaction significantly (p = .330 and 0.091 for GAD-7 and LSIZ, respectively). No interactions between intervention group and time of measurement were observed.

**Table 3:** Changes and comparison of anxiety, life satisfaction and pain at admission (t0), before surgery (t1) and before discharge (t2)

Outcome		Mean ± SD		
		Baseline (t0)	t1	t2
**GAD-7**	**IG**	3.98 ± 3.27	3.47 ± 2.58	2.18 ± 1.21
	**CG**	3.93 ± 3.20	4.04 ± 3.08	2.87 ± 2.00
**LSIZ**	**IG**	6.56 ± 1.70	8.27 ± 1.94	9.84 ± 2.09
	**CG**	6.67 ± 2.09	6.62 ± 2.08	9.02 ± 2.15
**VAS**	**IG**	NA	NA	4.16 ± 2.20
	**CG**	NA	NA	4.69 ± 2.26

Abbreviations: SD, standard deviation; IG: intervention group; CG: control group;

GAD-7: Generalized Anxiety Disorder 7-item Scale; LSIZ: Life Satisfaction Index Z scale; VAS: Visual Analogue Scale; NA: not applicable

**Table 4:** Estimated outcomes of mix linear model by intervention, time, and their interactions

Parameter	p	Estimation of difference (95% CI)
GAD-7: AIC = 1089.692, BIC = 1125.676		
Group IG to CG	0.330	-0.622(-1.884~0.640)
Time t0 to t2	< 0.001^*^	1.067(0.521~1.612)
Time t1 to t2	< 0.001^*^	1.778(0.710~1.645)
Interaction IG*t0	0.090	0.667(-0.105~1.438)
Interaction IG*t1	0.894	0.444(-0.617~0.706)
LSIZ: AIC = 1113.972, BIC = 1149.956		
Group IG to CG	0.091	0.822(-0.135~1.780)
Time t0 to t2	< 0.001^*^	-2.356(-3.041~-1.670)
Time t1 to t2	< 0.001^*^	-2.400(-3.060~-1.740)
Interaction IG*t0 to others	0.059	-0.933(-1.903~0.036)
Interaction IG*t1 to others	0.084	0.822(-0.112~1.756)

Abbreviations: CI, confidence interval; GAD-7: Generalized Anxiety Disorder 7-item Scale; LSIZ: Life Satisfaction Index Z scale; IG: intervention group; CG: control group

A stratified analysis of patients with/without generalized anxiety disorder (GAD-7 ≥ 10/ < 10) was also conducted regarding anxiety, pain, and life satisfaction in the CG and the IG. As shown in Table 5, the levels of anxiety (GAD-7: IG 11.33 ± 1.03 vs. CG 11.40 ± 1.14, p = .921, Cohen’s d = -0.064) and life satisfaction (LSIZ: IG 6.00 ± 1.90 vs. CG 6.00 ± 2.12, p = 1.000, Cohen’s d = 0.911) in the IG and the CG were quite similar at t0. A numerical higher improvement of anxiety was detected in the IG rather than the CG at t2 (GAD-7: IG 3.50 ± 1.22 vs. CG 6.80 ± 2.05, Cohen’s d = -1.956; LSIZ: IG 7.17 ± 0.98 vs. CG 5.80 ± 3.03, Cohen’s d = 0.608). As shown in Table 6, for patients without generalized anxiety disorder (GAD-7 < 10), the outcomes of varied parameters to variables were similar to the overall outcomes mentioned above. However, for patients with generalized anxiety disorder (GAD-7 ≥ 10), the parameter IG to CG and the interaction IG*t0 could cause a statistically significant difference to GAD-7 level, and none of intervention group, time of measurement, or their interactions showed this effect to LSIZ. Due to the fact that the interaction IG*t0 could create a statistically significant difference to GAD-7 level for participants with initial GAD-7 ≥ 10, the individual influence of intervention group in GAD-7 were further analyzed (Table 5), and it indicated that the GAD-7 level in t2 for IG group was significantly lower than that for CG group (p = .017). Mixed linear model was also used to analyze the influence of individual time of measurement in anxiety for either IG or CG group (Table 5), and the outcome showed time of measurement could cause significant difference in IG but not CG group. Moreover, compared with the overall outcome of pain, stratified analysis showed significant lower VAS scores in the IG than the CG (p = .017) at t2 for patients with initial GAD-7 ≥ 10, but not in those with initial GAD-7 < 10 (p = .279).

**Table 5:** Stratified analysis of patients with generalized anxiety disorder (GAD-7 ≥ 10). Changes and comparison of anxiety (GAD-7), life satisfaction (LSIZ) and pain (VAS).

Outcome		Baseline (t0)	t1	t2	p value
		Mean/Value			
**GAD-7**	IG	11.33 ± 1.03	8.17 ± 1.60	3.50 ± 1.22	< 0.001^*^
	CG	11.40 ± 1.14	10.20 ± 1.64	6.80 ± 2.05	0.071
	p	0.931	0.126	0.017^*^	
**LSIZ**	IG	6.00 ± 1.90	7.00 ± 1.67	7.17 ± 0.98	NA
	CG	6.00 ± 2.12	5.40 ± 2.19	5.80 ± 3.03	NA
**VAS**	IG	NA	NA	4.50 ± 1.76	NA
	CG	NA	NA	7.00 ± 1.00	NA
	p	NA	NA	0.017^*^	

**Table 6:** Stratified analysis of patients with/without generalized anxiety disorder (GAD-7 ≥ 10). Estimated outcomes of mix linear model by intervention, time, and their interactions

Parameter	GAD-7 ≥ 10		GAD-7 < 10	
	p	Estimation of difference (95% CI)	p	Estimation of difference (95% CI)
GAD-7	AIC = 120.160, BIC = 135.126		AIC = 713.836, BIC = 748.516	
Group IG to CG	0.001^*^	-3.300(-4.955~-1.645)	0.326	-0.401(-1.207~0.406)
Time t0 to t2	< 0.001^*^	4.600(3.631~5.569)	< 0.001^*^	0.625(0.357~0.893)
Time t1 to t2	< 0.001^*^	3.400(1.825~4.975)	< 0.001^*^	0.900(0.552~1.248)
Interaction IG*t0	< 0.001^*^	3.233(1.825~4.975)	0.201	0.247(-0.134~0.628)
Interaction IG*t1	0.221	1.267(-0.866~3.399)	0.601	-0.131(-0.626~0.364)
LSIZ	AIC = 143.952, BIC = 158.917		AIC = 959.202, BIC = 993.883	
Group IG to CG	0.428	1.367(-2.190~4.923)	0.059	0.831(-0.332~1.696)
Time t0 to t2	0.858	0.200(-2.153~2.553)	< 0.001^*^	-2.675(-3.348~-2.002)
Time t1 to t2	0.707	-0.400(-2.681~1.881)	< 0.001^*^	-2.650(-3.296~-2.004)
Interaction IG*t0 to others	0.372	-1.367(-4.553~1.820)	0.054	-0.940(-1.898~0.170)
Interaction IG*t1 to others	0.871	0.233(-2.855~3.322)	0.068	0.855(-0.064~1.774)

Abbreviations: CI, confidence interval; GAD-7: Generalized Anxiety Disorder 7-item Scale; LSIZ: Life Satisfaction Index Z scale; IG: intervention group; CG: control group

## Discussion

Our results show that discourse-based psychological intervention is an applicable method to improve perioperative levels of anxiety and life satisfaction and promote better post-surgical painful conditions for patients with AIS, especially those with moderate and severe anxiety symptoms (GAD ≥ 10). This corroborates the view that psychological intervention via discourse analysis is an effective method to manage emotional disorders [27] and alleviate the anxiety caused by diseases and operations [16–18, 20]. To our knowledge, the present study is the first to test the effectiveness of discourse-based psychological intervention on the alleviation of patients’ perioperative anxiety and surgery-related outcomes in AIS.

As shown in the current study, the preoperative anxiety level of the IG patients decreased after psychological intervention, and their emotional state remained at a low level after surgery. For the CG patients, the alleviation of anxiety was not observed until the corrective operation was completed, although it was still worse than that of the IG patients. Based on the stratified analysis of the patients with GAD-7 ≥ 10, it was shown that there were significant between-group differences on the improvement of anxiety condition, which indicated the effectiveness of psychological intervention for patients with moderate and severe anxiety issues. Moreover, the stratified analysis informed that the IG patients’ anxiety levels were raised significantly at t2 (p < .001), while that enhancement was absent in the CG patients (p = .071). For the patients with GAD-7 ≥ 10, the influence of Group IG to CG, time of measurement (Time t0 to t2 and Time t1 to t2), and their interactions (Interaction IG*t0 to others and Interaction IG*t1 to others) were all tested statistically insignificant (p > .05) in LSIZ by mixed linear models, therefore, detailed p values between IG and CG at varied time should not be further calculated individually.

These results highlight the advantages of discourse-based psychological intervention, in which more insightful, multiple perspectives are introduced for patients’ inner needs, emotions, and concerns, some of which are even ignored by patients themselves. With a better understanding of their worries and concerns, a more problem-oriented, empathic conversation can be established between patients and medical staff and a more inclusive and reasonable attitude towards symptoms of illness and related mental issues can be obtained by patients.

Pain levels in both groups were measured on the day before discharge. The data showed that the score of pains in the IG was lower than the CG. This difference was presented significantly in the stratified analysis, which indicated that for the patients with high-leveled anxiety (GAD-7 ≥ 10), the discourse-based psychological intervention could indirectly render the alleviation of post-surgical pain, since patients’ pain levels were argued to correlate with emotional status to some extent [28]. In contrast, psychological intervention had limited influence on post-surgical pain reduction for the patients with low levels of anxiety at baseline. Therefore, additional post-surgical coping strategies or medical interventions are needed in that post-surgical pain is mainly caused by surgery wounds [29] and affects patients’ functional recovery immediately during the inpatient stay [30].

As for life satisfaction, it was shown that both the IG and the CG enjoyed improvements after surgery. In previous studies, life satisfaction was argued to have negatively association with disease progression among AIS patients [31] and this situation was tested to be significantly improved at 1-year follow-up visit after corrective surgeries, when compared with AIS patients with non-surgical treatments [26]. The current research filled the gap on the study of AIS patients’ life satisfaction during perioperative period. It was worth noting that after psychological intervention, the life satisfaction of inpatients in the IG group, rather than CG group, improved significantly, which proved the effectiveness of discourse based psychological intervention in improving the life satisfaction of inpatients.

In the present study, the discourse analysis of participants’ narrations revealed certain consistency on their concerns and worries. Adolescents were most concerned about the physical aesthetics and physical functions after surgery, such as surgical risk, scars, deformity correction and sports participation. Participants tended to express their helplessness, embarrassment and frustration about their future life through negative evaluations, such as *mei* or *bu* (not/no/without), irregular and constant pausing or repetition, and application of simile and metaphor, such as *xiang canjiren* (like the disabled). It was proved that psychological intervention was able to effectively enhance patients’ satisfaction with themselves and life, as it was constructed on patients’ personal concerns and worries and made attempt to convey the concept of the “normal range” of health and beauty with empathy and comfort.

This study extended the application of discourse-based psychological intervention to AIS by showing the effect of reducing anxiety in AIS patients. In addition to the effectiveness of mental health in AIS patients, this study also proposed the correlation between pre-surgical anxiety level and post-surgical pain. Besides, this intervention has proved to be effective in improving the life satisfaction of AIS patients, although the directness of this assist or impact is open for further discussion, because the reduction of pain and anxiety levels may also help improve the life satisfaction of patients [32]. Apart from the above-mentioned effectiveness for AIS patients, discourse analysis per se was regarded as a new qualitative method to study and reveal patients’ real psychological conditions, which has never been applied in AIS research. In the current study, the topics and problems most concerned by AIS patients in China were highlighted and analyzed. These data are regarded as valuable resources for further research and investigation.

Our study has some limitations. To begin with, non-randomized selection of groups, small sample size and relatively short observing period limited our study. Based on this relatively small sample size, it is a bit difficult to discuss the impact of other confounding factors, such as gender, income and education, on patients’ anxiety, pain and life satisfaction; and therefore, we adopt the PSM analysis method so as to make the above confounding factors comparable between IG and CG. Since the entire study was conducted at the same hospital with limited number of patients, site-specific effects could not be excluded and the mental status of participants might not represent the whole population of AIS patients, whether national or cross-cultural. It may be interesting to see the effects of discourse-based psychological intervention on surgery-related anxiety, pain and life satisfaction in a larger portion of participants with diverse cultural backgrounds in a longer term. Besides, the correlation between pre-surgical psychological intervention and post-surgical life satisfaction is still inconclusive and needs further investigation. It is possible that other factors apart from psychological intervention may contribute to the improvement of post-surgical life satisfaction in the IG and the CG [32–34]. Furthermore, in the current study individual interviews and discourse-based psychological interventions were conducted by linguists with psychological research background. Although the technology required for discourse analysis and intervention is basic and accessible to non-experts, there is still a lack of relevant personnel in China’s hospitals, and therefore, special funds and personnel are required for medical staff training.

## Conclusions

Discourse-based psychological intervention can effectively reduce AIS patients’ perioperative anxiety, improve their life satisfaction, and alleviate the post-surgical pain of patients, especially for those with high-leveled pre-surgical anxiety.

## Data Availability

The datasets used or analyzed during the current study are available from the corresponding author on reasonable request.

## List of Abbreviations

AIS: Adolescent Idiopathic Scoliosis
IG: Intervention group
CG: Control group
PSM: Propensity score matching
CHD: Coronary heart disease
GAD-7: Generalized Anxiety Disorder 7-item Scale
LSIZ: Life Satisfaction Index Z scale
VAS: Visual Analogue Scale
